# Economic burden of breast cancer in northern Serbia

**DOI:** 10.3389/fpubh.2023.1265301

**Published:** 2023-12-15

**Authors:** Marko Milovic, Tatjana Tamas, Veljko Crnobrnja, Milica Paut Kusturica

**Affiliations:** ^1^University of Novi Sad, Faculty of Medicine Novi Sad, Novi Sad, Serbia; ^2^Oncology Institute of Vojvodina, Novi Sad, Serbia; ^3^Clinical Center of Vojvodina, Novi Sad, Serbia

**Keywords:** breast neoplasms, cost of illness, prevalence, health expenditures, hospital costs, Serbia

## Abstract

**Introduction:**

Breast cancer is the most common cancer in terms of incidence and mortality among all cancers in women in Vojvodina, the northern region of Serbia. In addition to the effectiveness and safety of therapy, it is important to put emphasis on the cost of treatment, as well as on the optimal allocation of limited resources.

**Objectives:**

This study aimed to assess the overall economic burden of breast cancer in Vojvodina, as well as the ratio of direct and indirect costs in 2019.

**Materials and methods:**

Costs were estimated using Cost of Illness (COI) evaluation, from a social perspective, based on the prevalence of the disease. The total costs included both direct and indirect expenditures. Direct costs associated with breast cancer comprised expenses linked to screening, hospital treatment, outpatient care, and prescribed medications. Indirect costs were estimated using a human capital approach, encompassing expenditures tied to lost productivity arising from sick leave, early retirement, and premature death.

**Results:**

The total cost of breast cancer in Vojvodina during 2019 was estimated to be 15 million euros. Among the total cost, direct costs accounted for 5 million euros, representing 34% of the overall expenses. Hospital treatment costs accounted for 76% of the direct costs, while screening costs represented 1%. Indirect costs amounted to 10 million euros, constituting 66% of the total cost. The primary driver was attributed to production losses caused by premature retirement, which accounted for 50% of the indirect costs.

**Conclusion:**

Breast cancer is a huge financial burden on both the health system and society in Vojvodina, accounting for 0.12% GDP. The dominance of indirect costs in total costs, can provide significant guidance to decision-makers in the healthcare system in terms of better allocation of limited resources to breast cancer prevention and early detection strategies.

## 1 Introduction

Breast cancer is defined as a malignant growth that occurs when breast tissue cells lose their characteristics of healthy cells and begin to grow and divide uncontrollably due to genetic and epigenetic factors (1). According to the International Agency for Research on Cancer (IARC), breast cancer is the most common malignant tumor worldwide, with 2.3 million new cases globally in 2020. Serbia is classified among countries with a high incidence rate of breast cancer (86.8/100,000 population). Globally, breast cancer is the leading cause of mortality among all cancers in women, accounting for 685,000 deaths in 2020 (2). Transitioning countries exhibit a 17% higher mortality rate compared to developed countries (3). Serbia holds the top position in Europe based on the standardized mortality rate (23.9/100,000) (2). In Serbia, there are two Institutes of Oncology, one located in Vojvodina, and the other in Belgrade. According to the data from the Institute of Public Health of Vojvodina (IPHV), breast cancer was the most common cancer in terms of incidence (22.7%) and mortality (16.6%) among all cancers in women in Vojvodina in 2019. Each year, over 1,200 women are diagnosed with breast cancer in Vojvodina, and approximately 500 women die from this disease (4). The increase in incidence, the emergence of innovative therapies, and the improvement in survival are the reasons for the constant rise in the economic burden of breast cancer, both for the healthcare system and society. Considering the high costs of breast cancer in the European Union, estimated at around 15 billion euros (0.15% of GDP), ranking second in total costs for all cancers, it is clear that the diagnosis, treatment, and monitoring of patients are of enormous importance for the healthcare system and the economy (5). The European Cancer Information System has recognized the lack of available cost data as one of the most critical barriers in the health planning process (6). The growing need for efficient resource utilization has made pharmacoeconomic studies increasingly significant as a tool that aids in decision-making regarding resource allocation in the healthcare system, particularly in the field of oncology. For the analysis and comparison of hospital costs, diagnostic-related groups (DRGs) are often used, which represent a method of classifying hospitalized patients into groups with similar clinical characteristics and requiring similar consumption of hospital resources. Classification based on the DRG system allows for the comparison of cost volumes, taking into account the number and complexity of cases. It is necessary to accurately define the major diagnostic category (MDC) and accompanying diagnoses, which refer to complications and comorbidities. The MDC refers to the main reason for which the patient was admitted to hospital, while accompanying diagnoses relate to conditions that have led to the need for additional diagnostics, therapy, or longer hospitalization of the patient (7). In order to facilitate more efficient treatment and better allocation of resources among institutions, DRG systems are currently implemented in over 30 countries worldwide, including Serbia since 2018 (8). Determining the total costs of a disease shows how much society spends on a particular illness and how much society would save if the disease were adequately treated. Analyzing the relationship between direct and indirect costs helps prioritize areas for research and financial investment by highlighting areas of deficit and opportunities for cost reduction (9). Comprehensive studies on the economic burden of breast cancer have been conducted in many countries in Europe and around the world, while such research is scarce in Serbia. Therefore, the aim of this paper is to estimate the overall economic burden of breast cancer in Vojvodina, as well as the ratio of direct and indirect costs in 2019.

## 2 Materials and methods

The research on costs associated with the health of breast cancer patients in Vojvodina was conducted as a one-year retrospective-prospective cost of illness study. The Ethical Board of the Oncology Institute of Vojvodina (No. 4/20/2–3,489/2–4), the Ethics Committee of the Primary Health Center Novi Sad (No. 21/4–1), and the Ethics Committee for Clinical Trials of the Faculty of Medicine Novi Sad (No. 01–39/298/1) approved the study protocol.

### 2.1 Total costs

Total costs of breast cancer in Vojvodina in 2019, were assessed using Cost-of-Illness analysis, a method within pharmacoeconomic evaluation (10). Prevalence-based approach was used, following a top-down perspective (11). Since breast cancer predominantly affects women, this research focused on female patients diagnosed with breast cancer (12). All patients from Vojvodina who were alive on December 31, 2019, with a diagnosis of breast cancer, as well as those who passed away in 2019 with this diagnosis, were considered. The total costs included both direct and indirect expenses. All costs were reported in prices for the year 2019, using the average exchange rate of the National Bank of Serbia (NBS) (1 euro = 117.8524 RSD) (13), as well as in the Purchase Power Parity (PPP) referenced to 27 EU countries in 2019 (PPP 1 euro = 60.95 RSD) (14).

#### 2.1.1 Direct costs

The direct costs of breast cancer included expenses related to screening, hospital treatment, outpatient care, and prescribed medications. When examining expenses related to a specific ailment using patient-level data, it becomes crucial to differentiate between the costs incurred by a patient with the said ailment and the overall cost of the ailment. Assessing healthcare expenses for patients with significant comorbidity and attributing all costs solely to the illness in focus may lead to an overestimation of the illness’s total cost. Achieving an accurate estimate for the cost of an ailment necessitates the identification and separation of costs specifically attributable to the studied illness from those caused by comorbidity. Therefore, only the costs directly linked to the studied illness should be included in the estimate to ensure precision. In this study, we focused on the cost caused by breast cancer. The resource consumption for patients with the main diagnosis of breast cancer can be assumed to have been caused by breast cancer, and would thus not have arisen if the patient had not had the illness. This method ignores the costs related to breast cancer as a secondary diagnosis. However, it includes the costs of other diseases in patients with breast cancer, which may have contributed to the resource use for patients with breast cancer as the first diagnosis. Since the two effects can be assumed to be rather small, and work in opposite directions, they are not expected to have any significant impact on the final result. This study focused on the cost of the additional resource consumption due to the main diagnosis of breast cancer. In theory, all relevant healthcare and non-healthcare costs should be included, but in practice, there is a limit to what can be identified and measured. Only resource use that has been assigned ICD-10 code C50 or other breast cancer diagnosis were included (15).

#### 2.1.2 Screening

Data on the number of women screened for breast cancer in Vojvodina in 2019 were obtained from the Institute of Public Health of Vojvodina (IPHV) (4). The contracted prices for breast cancer screening services and mammogram readings were taken from the Regulation on the Determination of Prices for Health Services at the primary level of healthcare, issued by the National Health Insurance Fund (NHIF) for the year 2019 (16). The number of services was then multiplied by unit prices.

#### 2.1.3 Hospital treatment costs

Data on the number of hospitalizations and hospital days due to breast cancer in Vojvodina in 2019 were obtained from IPHV (4). Data on the costs of hospital treatment for breast cancer patients were collected from the electronic system of the Oncology Institute of Vojvodina (IOV). The costs of inpatient treatment and daycare were analyzed. Resources used for breast cancer were identified based on breast cancer being the primary diagnosis for resource consumption. The following DRG codes were used for this purpose: J62A (malignant breast disorders, major complexity), J62B (malignant breast disorders, minor complexity), J06Z (major procedures for breast disorders), J07Z (minor procedures for breast disorders), J11Z (other skin, subcutaneous tissue, and breast procedures), and J14Z (major breast reconstructions) (8). The costs of medications used for hospitalized patients were incorporated into the DRG costs. Hospitalization costs realized at IOV were considered as costs for the entire Vojvodina, given that IOV is the only tertiary institution for oncology patients in Vojvodina, where modern oncology therapy for breast cancer is exclusively provided.

#### 2.1.4 Outpatient care costs

Data on outpatient care costs were obtained from the electronic database of the Primary Health Center Novi Sad (PHC NS) and included services provided by all departments utilized by patients diagnosed with C50. PHC NS is the largest primary healthcare institution in Vojvodina, covering the territory of Novi Sad and Sremski Karlovci municipalities (383,997 inhabitants), which represents 21.58% of the population of Vojvodina (17). Since data from all health centers in Vojvodina were not available and the standardized incidence rates for breast cancer in the South Bačka District (63/100,000) do not vary more than 10% compared to the Vojvodina region (69/100,000), the data from PHC NS for outpatient care were proportionally extrapolated to the population of Vojvodina (18).

#### 2.1.5 Costs of prescribed medications

Data on the most commonly prescribed outpatient medications for breast cancer and the number of prescribed medications for the C50 diagnosis were obtained from the electronic system of PHC NS. The costs were then estimated based on IQVIA data on the volume and value of sales of those medications in Vojvodina in 2019 (19).

### 2.2 Indirect costs

The indirect costs of breast cancer included expenses related to lost productivity due to sick leave, early retirement, and premature death. To estimate the indirect costs, a human capital approach was used, which considers lost productivity as the period until the employee’s return to work in the case of temporary absence or as the lost years of life until the end of the working career in the case of early retirement or premature death (20).

#### 2.2.1 Sick leave costs

Data on the number and duration of sick leave due to the C50 diagnosis in 2019 were obtained from the electronic system of the PHC NS. Since data from all health centers in Vojvodina were not available and the standardized incidence rate for breast cancer in the South Bačka District (63/100,000) does not vary more than 10% compared to the Vojvodina region (69/100,000), the data from PHC NS for sick leave were proportionally extrapolated to the population of Vojvodina (18). The sick leave costs were then calculated by multiplying the total number of months of absence by the average gross salary of women in Vojvodina in 2019, according to data from the Statistical Office of the Republic of Serbia (SORS) (21).

#### 2.2.2 Early retirement costs

Since the National Pension and Disability Insurance Fund (PIO fund) does not keep records of the number of disability pensions by disease diagnosis, data on the number of new recipients of disability pensions due to all cancers were used (22). Based on data from the Institute of Public Health of Serbia (IPHS) on the frequency of breast cancer in the occurrence of all tumors in the general population, the number of women who became new recipients of disability pensions due to breast cancer in Serbia in 2019 was estimated (18). Data on the proportion of disability pensions in Vojvodina compared to Serbia, average age, and length of service of recipients were obtained from the PIO fund (22). The estimated number of lost productive years of life during which women would work if they had not entered early retirement was then multiplied by the average gross salary of women in Vojvodina (21).

#### 2.2.3 Premature death costs

The number of women who died from breast cancer in Vojvodina in 2019 was obtained from data provided by IPHV (4). It was assumed that the distribution of deaths from breast cancer by age in Vojvodina was equal to that in Serbia (18). The number of lost productive years of life due to premature death from breast cancer was calculated based on the remaining years until retirement for each working-age group, assuming an equal distribution across years within each group. Since not all women were employed at the time of death, the employment rate of women in each age group in Vojvodina was taken into account, according to data from SORS for 2019. The estimated number of lost productive years of life was multiplied by the average annual gross salary of women in Vojvodina in 2019 (21). The estimated costs due to early retirement and premature death were discounted at a rate of 3%, while a sensitivity analysis tested discount rates of 0 and 5% (23).

## 3 Results

### 3.1 Direct costs

#### 3.1.1 Screening costs

In Vojvodina, a total of 18,779 women were screened for breast cancer during 2019, representing a coverage rate of 6.1% of the targeted age group of women aged 50–69. The contracted NHIF price for breast cancer screening in 2019 was 1.68 euros, while the contracted price for the mammogram reading service was 1.26 euros. By multiplying the number of services by the respective prices, the costs of breast cancer screening in Vojvodina in 2019 were estimated at 55,225 euros (PPP 106,783 euros).

#### 3.1.2 Hospital treatment costs

During 2019, breast cancer caused 3,361 hospitalizations in Vojvodina, ranking it fifth among the leading diagnoses for hospitalization of women in Vojvodina and first among all tumors. At IOV, there were 2,503 episodes of hospital treatment for breast cancer in 2019, with costs amounting to 3.84 million euros (PPP 7.42 million euros). The costs of medications were incorporated into the hospital treatment costs and amounted to 2.68 million euros (PPP 5.18 million euros). Medications accounted for 70% of the total hospital treatment costs and were most prevalent in the J62A (81%) and J62B (91%) groups, while their contribution to other groups related to surgical procedures was much lower. J62A had the highest share in costs among all DRGs (60.4%), followed by J62B with a share of 22.1%, and J06Z with a share of 16.6% of the total costs. The share of other DRGs was below 1% (Table 1).

**Table 1:** DRG costs (EUR).

DRG code	DRG group	Cost of medications	Total hospital costs	%
J06Z	Major procedures for breast disorders	21,612	638,083	3%
J07Z	Minor procedures for breast disorders	1,063	24,118	4%
J11Z	Other skin, subcutaneous tissue, and breast procedures	2,640	9,449	28%
J14Z	Major breast reconstructions	53	2,842	2%
J62A	Malignant breast disorders, major complexity	1,878,810	2,319,488	81%
J62B	Malignant breast disorders, minor complexity	773,661	847,034	91%
Total		2,677,840	3,841,014	70%

Unspecified malignant neoplasm of the breast (C50.9) had the highest share in costs among all ICD-10 codes (48.5%), followed by malignant neoplasm of upper-outer quadrant of breast (C50.4) with a 33.3% share of the total costs. The share of other groups was below 10%.

#### 3.1.3 Outpatient care costs

During 2019, a total of 2,480 patients diagnosed with C50 utilized 138,852 outpatient services at the PHC NS. The total cost of services amounted to 154,664 euros. The highest cost was incurred in the laboratory diagnostics service (65,954 euros, 43%), followed by the general medicine service (39,382 euros, 25%), and the home treatment service (19,891 euros, 13%). Extrapolation estimated that outpatient care costs in Vojvodina in 2019 due to breast cancer amounted to 716,701.4 euros (PPP 1.38 million euros).

#### 3.1.4 Costs of prescribed medications

The most commonly prescribed outpatient medications for breast cancer were estrogen receptor antagonists (tamoxifen) and aromatase inhibitors (anastrozole, letrozole, and exemestane). In Vojvodina, during 2019, 80,839 packages of these medications were consumed, with a value of 441,763 euros (PPP 0.85 million euros).

The direct costs of breast cancer in Vojvodina in 2019 were estimated at 5.05 million euros (PPP 9.77 million euros). Hospital treatment costs accounted for 76% of the direct costs, while screening costs represented 1% of the direct costs.

### 3.2 Indirect costs

#### 3.2.1 Sick leave costs

According to data obtained from the PHC NS for 2019, 182 patients utilized sick leave due to breast cancer, with an average duration of 124 days, totaling 752.3 months. When extrapolating PHC NS data to Vojvodina, the total duration of sick leave was estimated at 3,486.1 months. Based on the average monthly gross earnings of women in Vojvodina in 2019, which amounted to 560.54 euros, it was estimated that the costs due to temporary work absence were 1.95 million euros (PPP 3.78 million euros).

#### 3.2.2 Early retirement costs

In 2019, the number of new pension beneficiaries in Serbia was 84,691, of which 13,720 (16.2%) were new recipients of disability pensions. Tumors accounted for 29.5% of disability pension cases, resulting in 4,047 individuals going into disability retirement due to tumors. The prevalence of breast cancer among all tumors in the total population was 11.2%, so it was estimated that 453 individuals went into disability retirement due to breast cancer in Serbia in 2019. In Vojvodina, 23.6% of disability pensioners were accounted for, resulting in an estimated 107 women going into disability retirement due to breast cancer in Vojvodina during 2019. Women who went into disability retirement had an average work experience of 21 years, compared to the average work experience of 30 years for regular retirement. Therefore, each woman lost an average of 9 productive years of life, totaling 963 years. When the number of lost productive years of life was multiplied by the average annual gross earnings of women in Vojvodina in 2019, and by applying a discount rate of 3%, the costs of early retirement due to breast cancer in 2019 in Vojvodina were calculated to be 4.96 million euros (PPP 9.6 million euros).

#### 3.2.3 Costs of premature death

In Vojvodina, 444 women died due to breast cancer in 2019, accounting for 26.7% of breast cancer-related deaths in Serbia. Since data on the number of deceased working-age women by age groups in Vojvodina were not available, these were calculated by proportionally reducing available date for Serbia by 26.7%. The average age at which women entered retirement in 2019 was 62 years. For each age group, taking into account the employment rate, the number of lost productive years of life due to breast cancer was estimated. The total number of lost productive years due to breast cancer in Vojvodina was estimated at 709 years. After multiplying the number of lost years by the average earnings of women in Vojvodina in 2019 and applying a discount rate of 3%, the costs of premature death due to breast cancer in 2019 in Vojvodina were estimated at 3.03 million euros (PPP 5.85 million euros). The costs of premature death were highest in the age group of women aged 50 to 54, accounting for 28% of the total costs. The lowest costs were in the age group of women aged 25 to 29, representing 2% of the total costs (Table 2).

**Table 2:** Costs of premature death.

Age group	Number of women deceased from breast cancer	Employment rate	Lost productive years of life	Discounted costs of mortality (EUR)*
25–29	1	64%	24	57.453
30–34	2	64%	46	128.450
35–39	6	72%	100	321.702
40–44	8	72%	114	426.218
45–49	17	67%	165	712.672
50–54	26	67%	172	861.717
55–59	37	36%	68	394.194
60–64	51	36%	19	124.690
			709	3.027.094

*Discount rate 3%.

The indirect costs of breast cancer in 2019 in Vojvodina were estimated at 9.95 million euros (PPP 19.23 million euros), and they accounted for 66.3% of the total costs of breast cancer. Half of the indirect costs were due to premature retirement, while 30% were due to premature death.

The total costs of breast cancer in 2019 in Vojvodina were estimated at 15 million euros (PPP 29 million euros). The largest share in the total costs was accounted for by costs of premature retirement (33.1%) and hospitalization costs (25.6%) (Table 3). The estimated total costs of breast cancer in Vojvodina amounted to 0.12% of the regional GDP.

**Table 3:** Total costs of breast cancer.

Costs	EUR	%
Screening costs	55,225.23	0.4%
Hospital treatment costs	3,841,013.53	26%
Outpatient care costs	716,701.44	5%
Costs of prescribed medications	441,763.00	3%
Direct costs	5,054,703.20	34%
Sick leave costs	1,954,069.38	13%
Early retirement costs	4,964,467.24	33%
Costs of premature death	3,027,094.49	20%
Indirect costs	9,945,631.10	66%
Total costs	15,000,334.30	100%

### 3.3 Sensitivity analysis

By applying discount rates of 5 and 0%, there were changes in the costs of premature retirement and death, which affected the indirect and total costs of breast cancer.

**Figure 1:**
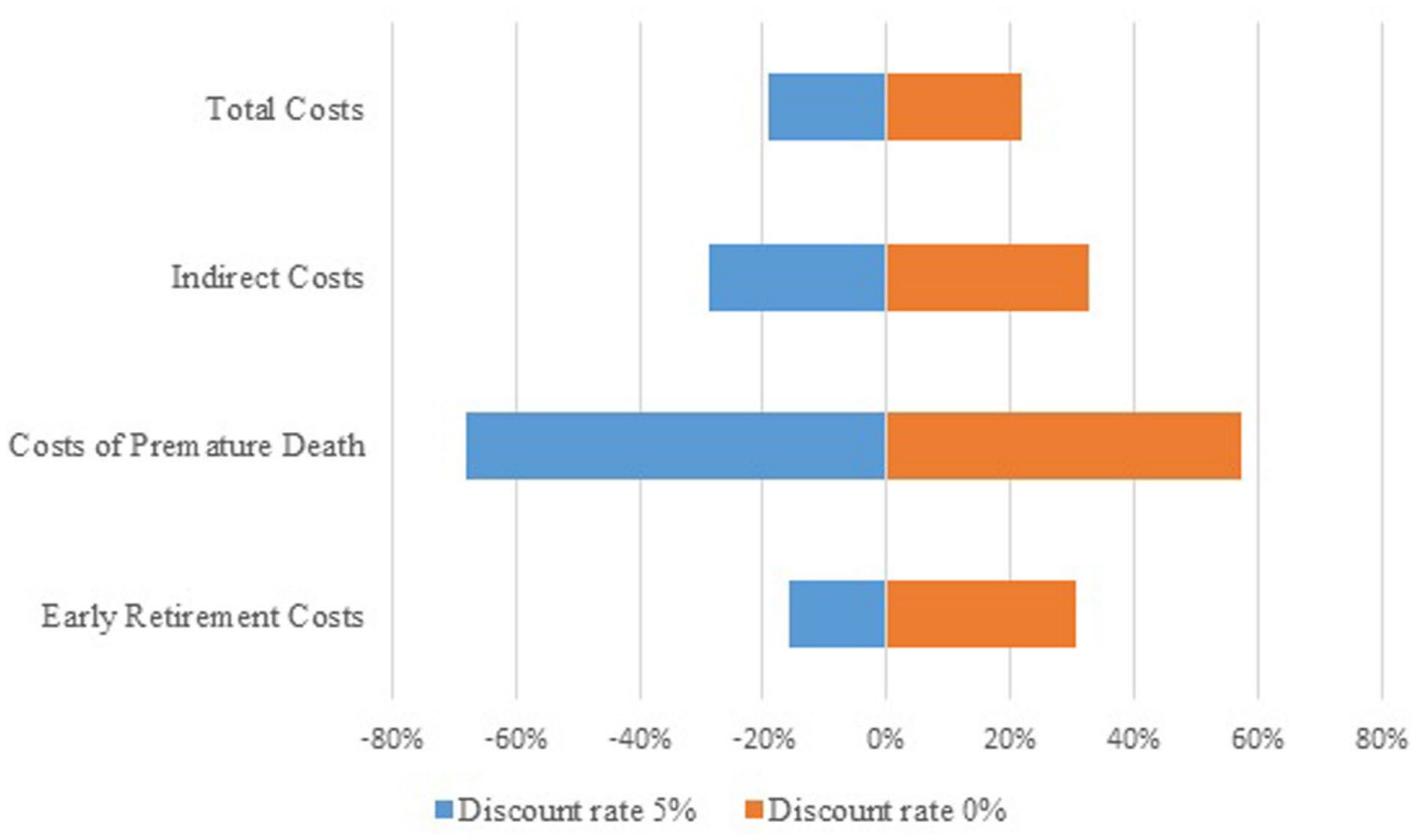
Tornado diagram.

The costs of premature death were the most sensitive to changes in the discount rate. When applying a discount rate of 5%, the costs were lower by 68%, while when applying a discount rate of 0%, they were higher by 57%. The total costs, in the first scenario, were lower by 19% with the change in the discount rate, and in the second scenario, they were higher by 22%, compared to the application of a 3% discount rate (Figure 1).

## 4 Discussion

Previous scientific papers in Serbia have only analyzed specific segments of direct or indirect costs of breast cancer. Bencina et al. (24) estimated the indirect costs due to mortality in Serbia to be 18 million euros in 2019. A doctoral dissertation on the economics of cancer in central Serbia estimated the average direct medical costs of breast cancer to be 24,000 euros per patient, with medications being the largest cost driver (25). In a study analyzing costs in the terminal stage of cancer patients, Kovačević et al. (26) estimated the direct costs of breast cancer to be an average of 13,100 euros per patient, with medications also representing the largest share of direct costs. Given the significant amounts of direct and indirect costs associated with breast cancer, it was necessary to conduct a comprehensive analysis of total costs, in order to contribute to informed decision-making regarding the allocation of limited healthcare resources.

The study examined the total costs of breast cancer from a societal perspective, based on disease prevalence, in line with the practice of conducting cost-of-illness studies in other countries (5, 27–36). The study encompassed the costs of breast cancer in 2019 to eliminate the impact of the COVID-19 pandemic, which was declared in 2020 and significantly affected all processes in the healthcare system.

The total costs of breast cancer in 2019 in Vojvodina were estimated at 15 million euros, accounting for 0.12% of the regional GDP. This result is consistent with studies conducted worldwide that included both direct and indirect costs, where the percentage of total costs of breast cancer as a share of GDP ranged from 0.06% in Russia (28) to 0.19% in Iran (33). Total costs in many studies have been estimated within this range of GDP, including California, USA (0.08% of GDP) (32), South Korea (0.09% of GDP) (34), Sweden (0.11% of GDP) (27), Japan (0.11% of GDP) (35), the Netherlands (0.14% of GDP) (29), and 27 EU countries (0.15% of GDP) (5).

The direct costs of breast cancer were estimated at 5.05 million euros, accounting for 33.7% of the total costs, which is in line with the results of studies conducted in Sweden (30%) (27) and Russia (37%) (28). The analysis included direct medical costs of screening, hospitalization, outpatient care, and prescribed medications, following the model of the Swedish study (27). Most studies conducted in other countries also included costs of hospital and outpatient care as well as medications but did not include screening costs (5, 32–36).

Screening costs in Vojvodina accounted for only 1% of the direct costs, which is significantly lower than the Swedish study where screening costs represented 22% of the direct costs (27). This result may be due to the higher cost per person for screening in Sweden (31.9 euros) compared to Vojvodina (2.9 euros), as well as a significantly higher percentage of women participating in the screening program compared to the population in Sweden (7% vs. Vojvodina’s 1%). In Vojvodina, 18,779 women were screened for breast cancer in 2019, representing a coverage rate of 6.1% of the target age group of women aged 50 to 69. If Vojvodina achieved the planned coverage rate of 50% of the target age group, which included 309,724 women, the costs would amount to 449,100 euros, still representing less than 10% of the direct costs (4).

The notably small proportion of costs allocated to screening in Vojvodina raises important public health considerations. Enhancing screening participation rates, aligning with the planned coverage rate of 50% for the target age group, could not only contribute to earlier diagnoses but also prove cost-effective in the long run. Addressing barriers to screening accessibility and awareness campaigns may be crucial for public health authorities and policymakers aiming to improve breast cancer outcomes in Vojvodina.

The costs of hospitalization amounted to 3.84 million euros and accounted for 76% of the direct costs of breast cancer, which is consistent with the results of studies conducted in California (67%) (32) and the Netherlands (78%) (29). The analysis included only costs where the primary cause of hospitalization was a diagnosis of C50 to avoid overestimating costs due to comorbidities. This method excluded costs of breast cancer as a secondary diagnosis but included costs of other diseases as secondary diagnoses, which to some extent contributed to resource consumption. Since these two factors act in opposite directions and offset each other, we do not expect them to have a significant effect on the final result.

Medications for breast cancer therapy are mostly administered in a hospital setting (37). The costs of hospital medications were included in the hospitalization costs and accounted for 70% of the costs, which is comparable to the results of the doctoral dissertation on the economics of cancer in central Serbia, where the share of medications in costs was 72% (25). Similarly, a study conducted in Russia also indicated a high share of medications (70%) in direct costs (28).

Six DRG codes were included in the analysis, with the highest costs observed in groups J62A and J62B due to the highest medication usage in these groups. In contrast, DRG codes related to malignant breast disorders major or minor complexities accounted for only 30% of hospitalization costs in the Swedish study, while DRG codes for surgical procedures had the highest share (27). The highest costs for hospitalization (excluding unspecified breast cancer) were associated with the C50.4 code (malignant tumor of the upper outer quadrant of the breast), accounting for one-third of the total costs. This result is in line with the fact that breast cancer most commonly occurs in the upper outer quadrant of the breast (38).

The costs of outpatient care, after hospitalization costs, had the largest share in direct costs and were estimated at 716,000 euros (14%). Lidgren et al. (27) estimated a significantly higher share of outpatient care costs in direct costs (32%), but they included costs of hospital outpatient visits in this category, while in our study, those costs were included in hospital costs. The laboratory diagnostics service (43%) had the highest share in outpatient care costs, followed by the general medicine service (25%). The home care service ranked third (13%), indicating a significant number of patients who were unable to visit outpatient clinics. A study conducted in California similarly estimated the share of home care costs (19%), with outpatient physician visits being the largest cost driver (32).

By examining electronic data from the PHC NS on the number of prescribed prescriptions for C50 diagnoses and consulting with experts, it was concluded that the most commonly prescribed ambulatory medications were tamoxifen, anastrozole, letrozole, and exemestane. These medications belong to the group of drugs used to treat HR+ breast cancer, which is the most common type of breast cancer (39). Ambulatory prescribed medications were estimated to account for 9% of the direct costs, which aligns with the results of Lidgren et al. (27).

The concept of indirect costs is still a topic of discussion in terms of what they include and how they are estimated. A comprehensive definition of indirect costs refers to the lost productivity due to morbidity (temporary or permanent absence from work) and mortality (premature death) (9). Studies often analyze only the indirect costs of morbidity (28, 40) or only the costs of mortality (24, 32, 33), while our study included both groups of indirect costs, following studies conducted in Sweden, Spain, South Korea, and Japan (27, 30, 34, 35). The estimation of indirect costs in our study was based on the human capital theory approach, which is commonly used in cost-of-illness studies, including many studies that have analyzed the costs of breast cancer (27–29, 31–35). Critics of the human capital approach point out that individuals who are temporarily absent from work can compensate for lost productivity upon returning from sick leave, and that their colleagues can substitute for them in certain tasks during their absence. In the case of early retirement or death, an unemployed person can replace a worker, as predicted by the replacement cost method, which is used as an alternative method and estimates indirect costs significantly lower than the human capital method (21). The authors of studies conducted in Spain and Russia estimated the indirect costs of breast cancer using both methods, with cost estimates based on the friction cost method accounting for only 4% of the human capital method costs in Spain (30) and 7% in Russia (28).

The indirect costs in Vojvodina were estimated at 9.95 million euros, accounting for 66.3% of the total costs of breast cancer, which is consistent with the results of studies conducted in California, Sweden, Iran, Japan, and Russia, where the share of indirect costs exceeded 60% (27, 28, 32, 33, 35). The reason for such high indirect costs of the disease is primarily the large number of working-age women who develop breast cancer (41).

The costs of morbidity due to temporary absence accounted for 20% of the indirect costs and were related to sick leave costs, in line with the methodology and results of the study conducted in Sweden (29%) (27). The costs of early retirement accounted for 50% of the indirect costs, which is comparable to the findings of studies conducted in Spain (55%) (30) and the Netherlands (52%) (29).

The costs of mortality accounted for 30% of the indirect costs, while the Swedish study had a significantly higher share of these costs (52%) (27). The lower costs of mortality in our study may be attributed to the inclusion of the employment rate in the cost estimation, as not all deceased women were employed. This methodology was adopted from studies that examined the costs of breast cancer mortality (24, 32–34).

The costs of mortality were highest in the age groups of 50–54 years and 45–49 years, despite the highest number of deceased women being in the age groups over 55 years. This is due to a higher number of lost productive years of life in younger women, as well as a higher employment rate. Daroudi et al. (33) also found that the highest costs due to premature death in Iran were primarily among younger women, with the highest costs observed in the age group of 45–49 years.

The costs of morbidity and mortality were discounted at a rate of 3% in line with the practice of cost estimation in pharmacoeconomic evaluations (9). By varying the discount rates from 0 to 5% in the sensitivity analysis, significant changes in indirect costs were observed, particularly in the costs of premature death. This is due to the significant influence of the discount rate when calculating the lost productive years of life, especially in younger individuals, considering the long discounting period. Kim et al. (34) also found a significant impact on breast cancer mortality costs through sensitivity analysis by varying the discount rates of 0, 3, and 5%.

### 4.1 Limitations of the study

In theory, a cost-of-illness study should encompass all medical and non-medical expenses related to the observed disease. However, in practice, there are limitations in identifying and measuring certain costs, leading us to exclude non-medical direct costs (patient transfers, informal care, childcare, etc.) as well as non-material costs such as suffering and pain, which are challenging to quantify monetarily.

Data from private laboratories and clinics regarding the number of tests and procedures utilized by breast cancer patients were not included in our research. It was estimated that these costs do not significantly impact the total cost amount.

Our analysis covered all outpatient costs of patients diagnosed with C50 in 2019 at the PHC of Novi Sad, even though some visits may not have been primarily related to breast cancer. The costs of outpatient prescribed medications did not include antiemetics, analgesics, and dietary supportive therapy, as it was not possible to assess their proportion of use among breast cancer patients.

Some data were not available specifically for Vojvodina, so an approximation of the data for Serbia had to be used.

Despite these limitations, which are typical in the field of health economics, this study is, according to available literature, the first comprehensive research on the total costs of breast cancer in Serbia and may serve as a basis for future investigations.

## 5 Conclusion

Breast cancer is a disease that poses a significant financial burden on both the healthcare system and society in Vojvodina. By conducting this research, a better understanding of the methodology for assessing the direct and indirect costs of breast cancer has been achieved, which will be used in future studies. The dominance of indirect costs in total costs, can provide significant guidance to decision-makers in the healthcare system in terms of better allocation of limited resources to breast cancer prevention and early detection strategies.

## Data availability statement

The raw data supporting the conclusions of this article will be made available by the authors, without undue reservation.

## Ethics statement

The studies involving humans were approved by the Ethical Board of the Oncology Institute of Vojvodina (No. 4/20/2–3,489/2–4), the Ethics Committee of the Primary Health Center Novi Sad (No. 21/4–1), and the Ethics Committee for Clinical Trials of the Faculty of Medicine Novi Sad (No. 01–39/298/1) approved the study protocol. The studies were conducted in accordance with the local legislation and institutional requirements. Written informed consent for participation was not required from the participants or the participants’ legal guardians/next of kin in accordance with the national legislation and institutional requirements.

## Author contributions

MM: Formal Analysis, Visualization, Writing – original draft, Writing –review & editing. TT: Data curation, Writing – review & editing. VC: Writing – review & editing. MPK: Methodology, Conceptualization, Writing – review & editing, Supervision.
